# Financial wellness awareness: A step closer to achieve Millennium Development Goals for Pakistan

**DOI:** 10.12669/pjms.311.6058

**Published:** 2015

**Authors:** Rehana Rehman, Shahjahan Katpar, Rakhshaan Khan, Mehwish Hussain

**Affiliations:** 1 Dr. Rehana Rehman, Assistant Professor, Department of Biological and Biomedical Sciences, Aga khan University, Karachi, Pakistan.; 2 Dr. Shahjahan Katpar, Professor Oral & Maxillofacial Surgery, Institute of Dentistry, Liaquat University of Medical & Health Sciences, Jamshoro, Pakistan.; 3 Dr. Rakhshaan Khan, Public Health Physician,; 4 Mehwish Hussain , Senior Lecturer of Biostatistics, Dow University of Health Sciences, Karachi, Pakistan.

**Keywords:** Financial wellness, Financial literacy, Financial Security, Medical students, Wellness wheel, Pakistan

## Abstract

**Objective:**

To explore financial wellness (FW) awareness amongst public and private sector medical college students of Karachi.

**Methods:**

A cross sectional questionnaire based survey was conducted on medical students from 3 public and 5 private sector medical colleges of Karachi from February 2011 to December 2011. All ethnic groups having age range of 18-23 years were included. A questionnaire tailored from wellness wheel evaluated the responses of FW on a four point Likert’s scale ranging from 0-3(never, sometimes, mostly, and always). Factor analysis explored common FW factors among both public and private sector medical college (MC) students.

**Results:**

Private MC Students were better in terms of making short and long terms financial goals compared to students in public sector. The students of public MC were more focused to make and restricting to given budgets (p=0.05). The FW element of keeping savings in bank account was responded more by private MC candidates (P < 0.0001) but was spent thrift as well (P < 0.0001). Factor analysis revealed two factors; ‘Financial Security Wellness’ which was better in Private MC Students (p=0.001) and ‘Care towards Expenses Wellness” in which results were not significant.

**Conclusion:**

Both groups of medical college students lacked FW awareness element in terms of caring towards financial expenses. The awareness of importance of financial security was practiced better by private MC students in terms of making short and long term financial goals and keeping savings in bank accounts. They were however deficient in the knowledge of making and restricting themselves to budgets.

## INTRODUCTION

The concept of wellness is not new to higher education, which may play a significant role in the academic achievements of an individual as well as adjustment to their surrounding environment.^1^^,^^2^ Wellness Wheel has different dimensions, namely: physical, emotional, intellectual, social, spiritual, environmental, financial and occupational that should be balanced and in the right proportion to give a feeling of normal well being, harmony and normal health.^3^ Financial Wellness (FW) is the knowledge and having control about personal finances that makes one feel satisfied in the existing situation and also means, one’s ability to mobilize finances in a foreseeable future situation.^1^

Recently, there has been a tremendous mushrooming growth in number of private sector medical colleges (Private MC) in Pakistan.^2^ The admissions in these institutes are based primarily on financial affordability of the candidate followed by their merit scores. On the contrary, in Public sector medical colleges (Public MC) domicile and open merit, in addition to better are taken deeper into account rather than huge fee amounts.^4^ The wellness awareness issues are important for students in both sets of medical institutions in our country, so as to train those as proficient health care providers who could take care of their wellbeing as the first and foremost priority, in addition to their professional responsibility.^5^

Financial wellness (FW) is ability to recognize the importance of sustaining ourselves financially stable for our short and long term goals. Financial literacy (FL) is the use of knowledge and skills to effectively manage financial resources for financial well-being.^6^ FL like health literacy is well documented by International Consumer World. It has no universal acceptable definition and can be grouped into two main domains: financial security and care towards expenses with understanding, using and application of finances.^7^ Majority of students in our country, including medical students rely and get their financial support from their parents, whereas in advanced countries, strong financial-loan support system exists for students.^8^ The awareness of FL can help in restoration of FW which in turn is a composite of wellness wheel. 

FW takes into account both the objective perceptions and subjective indicators of an individuals’ personal financial status. These objective indicators include awareness about: income, debt, savings, knowledge about financial products and services, planning ahead and staying on budget. Subjective indicator include: an individuals’ satisfaction derived from the capability to maintain financial balance in terms of where money comes from and where it’s going.^5^

Financial awareness and its practical utility should not be limited to medical students, rather should have a bigger spectrum and include students from all other health professional related fields like Dentistry and Pharmacy. The awareness of FL can help in restoration of FW which in turn is a composite of wellness wheel and may help in their overall performance. We feel it is completely unaddressed in our health profession as we have been unable to find any related local references and in order to fill the wide gaps present this topic has been selected by the authors. This study is another pillar of our Wellness wheel domain, related research, regarding the medical profession and health care delivery system and most probably will serve as a foundation pillar on this neglected area in Pakistan. The objective of the study is to explore FW in terms of economic understanding and financial utilization of available resources in both genders of private and public sector medical college students in Pakistan.

## METHODS

The questionnaire based survey was conducted after approval from Research & Ethical Committee of Bahria University Medical & Dental College, from February 2011 till December 2011. Self-administered questionnaire was distributed to three public and five private sector medical colleges who gave permission for the survey. The questionnaire was pretested and verified from error on group of fifty students. The reliability of the questionnaire was determined by measuring the related Cronback Alpha (Alpha=0.80). Sample size was based on a population of 3,000 with e (margin of error) of 5% and z (confidence interval) of 95%. Convenience sampling of 800 first year MBBS students (100 from each medical college) of either gender, all ethnic groups and age range 18-23 years was done with exclusion of foreign students. They were briefed with consent, about the objective of study, questions for evaluation of FW derived from Wellness wheel^6^ and 4 point Likert scale from 0-3 (never, sometimes, mostly, always) for giving in responses. 

***Statistical Analysis: ***Predictive Analysis Software (PASW 18.0 SPSS Inc. Chicago Illinois US) for windows was used for data interpretation. Descriptive analysis was given in frequencies (percentages) for categorical variable and mean ± standard deviation for continuous variables. FW total score was obtained by summation of individual response to each item. Kaiser-Meyer-Olkin (KMO) measure of sampling accuracy and Bartlett’s test of sphericity were calculated to check need of factor analysis. Eigen values criterion was used to extract factor and then common factors were formed with loadings more than 0.30. Mann-Whitney U test was used to compare different FW scores between students from public and private MC. P value less than 0.05 was set as level of significance of detecting differences.

## RESULTS

From the total 800 participating students, seven hundred and thirty six student’s responses were included in this study with four hundred fifty from private MC while two hundred and eighty six were from public MC. Female participants were more than male in both public (n = 234, 81.8%) and private (n = 292, 64.9%) medical colleges (P < 0.0001). The mean ages of students from public and private MC were 19.3 ± 0.84 years and 19.4 ± 1.11 years respectively (P = 0.088). Table-I displays the comparison between public and private medical college students. Short and long terms financial goals setting were always set by 30% (n = 217) participants. Forty percent (n = 291) of them budgeted their spending each month. Public MC Students restricted themselves to given budget more than private MC students (P = 0.056). Higher proportion of private MC candidates were used to spending whatever they get (P < 0.0001). Savings in bank accounts was reported lower by Public MC students (P < 0.0001).

The KMO was 0.624 and Bartlett’s test of accuracy was found to be significant (P < 0.001). Eigen value was more than 1 for two factors. Thus, these two factors were extracted and name based on value of loadings within each factor. Factor 1 was named ‘Financial Security Wellness’ which comprised of ‘making financial goals’ and ‘keeping savings in bank account’. It was significantly better in Private MC students (p=0.001). Factor 2 “Care towards Expenses Wellness” had highest positive loadings for ‘making budget’ and ‘restricting oneself to budget with high negative loading for ‘over spending’. Private MU students were slightly better in this aspect of FW, but results were not significant (Table-II).

## DISCUSSION

Health care delivery system providers in Pakistan, belong to both public and private sectors that play their respective professional roles, in variable proportions. Entrance into the respective health sector academic institutions is associated not only with the individual’s performance in the entrance examination and prior grades obtained but also on the family financial status, ethnicity, gender biasness and cultural-educational backgrounds.^7^ In the two different sets of medical colleges in our country, students join from diverse back grounds together with a small percentage of foreign students, whose quality of life and needs differ from the local inmates.^8^ We should remember that, the essence of medical education is to produce erudite, competent and skilled professionals who should play a vital role in promoting public health care from both sets of institutions.^9^

Financial debt related issues are more common in the West due to availability of loan facilities for students to pursue their educational careers. These debts are a consequence of strong capitalist culture, whereas students from eastern countries depend on financial support from the parents.^10^^,^^11^ It has been documented that demands and stresses of professional education in the absence of financial loan support system are more likely to affect behaviors and life styles.^5^^,^^12^ It is documented that financial education significantly improved student financial knowledge and maintenance of their bank accounts to save money for future needs.^13^^,^^14^ This knowledge about FW can thus help medical students to acquire financial security (FS) that will also help in balancing their academic performance and wellbeing at respective institutes.

FS is a broad term expressed in terms of: keeping a balance of income and expenditures, making and maintaining a bank account, making short and long term financial goals and saving some for the rainy days.^15^^,^^16^ In our study, factor FS was represented by making short and long term goals and maintaining bank accounts. Short term financial goals can be made by making an outline of list of objectives, amount to be spent on them on daily or weekly basis and plan of the whole month. This may include categories of payment of monthly fee, laundry expenditure, groceries charges, utility bills, hostel mess expenses and payment of servants especially for especially for hostilities and those who live in apartments outside hostels. Long term financial goals may include payments in specific months of the year like insurance payment, car or house loan payment, annual charges and miscellaneous.^10^ Our study showed that majority of students from both sets of medical institutes were not fully aware of the concept of FS, however Private MC students were better in terms of making financial goals and maintaining bank account.

In addition to FS, Financial literacy (FL) is the knowledge which enables financial planning by developing and implementing a budget with respect to short and long term financial goals.^17^ A budget is a schedule “to live within one's financial means” and is a pragmatic financial plan made on existing income and expenditure. Preparing (and sticking to) a budget is an essential component of responsible money management. It is based on an in-depth understanding of where and how much money needs to be spent and is made with the knowledge of available funds, list of expenditures with their priorities and at the time line financial independence**. **A budget forces to get spending under control and "live below your means". Creating a budget is an imperative step for determining financial health and sticking to it helps to pay off debts which augments FW. The money that is saved can be used to pay down the principal of debts, or kept into a savings or investment account, again for a rainy day. It was noticed in our study that, Public MC students were good in budgeting their financial accounts and Private MC students were in the habit of spending whatever they had and at the same time used to keep their savings in the bank.

Pakistan is the seventh most populous developing country and by 2050, is expected to be one of the largest in the world.^18^ Despite having a better per capita income, the utilization of health services in our country is threatened by present poor adult literacy rates and other associated factors^19^ therefore, we also need to address our national developmental goals^19^ Although our existing health education and delivery care system involves both Private and Public Sectors, yet we have not been able to achieve the millennium development goals(MDGs), due to numerous challenges.^20^ FL at individual and community level demands a corporate agenda so as to fulfill all national and associated provincial challenges.^21^ In our circumstances, students of Private MC are expected to be financially well off as compared to public MC who were found to be spent thrift and were not good at making budget. They however had FL of bank saving accounts to rescue their FS. The needs assessment focused on the control of expenses, can give a sigh of relief, a restful sleep at night with something saved for the rainy days. These steps will enable us to acquire an important spoke of wellness wheel^21^ which will be beneficial not only for our Physicians but also for economic well being of the country.

Since the instrument on FW was a smaller component of a bigger picture of wellness research sphere, this study had some limitations like lesser number of questions. In the absence of literature available on similar local research the validity of questions on FW could not be checked. Similarly, no questions on confounders were incorporated assuming that there is no previous exposure of medical students to financial education. Therefore, generalization of results may not be appropriate to other target population. The study however highlighted the need of creating FL among medical students of our country, irrespective of their financial backgrounds. The destination of FW can thus be acquired by FL with the help of transformational change by: budgeting, caring towards expenses, making financial goals and maintaining bank accounts.

**Table-I:** Comparison of financial dimensions of wellness in public and private medical students

		**Public** **N=286**	**Private** **N=450**	**Total**	**P Value**
I make both short & long term financial goals	Always	101 (0.35)	181 (0.40)	282 (0.38)	0.272
	Usually	85 (0.29)	132 (0.29)	217 (0.30)	
	Sometimes	50 (0.18)	80 (0.18)	130 (0.18)	
	Never	50 (0.18)	57 (0.13)	107 (0.14)	
I budget my spending each month	Always	122 (0.43)	169 (0.38)	291 (0.40)	0.400
	Usually	58 (0.20)	111 (0.25)	169 (0.23)	
	Sometimes	50 (0.17)	86 (0.19)	136 (0.18)	
	Never	26 (0.20)	84 (0.19)	140 (0.19)	
I restrict myself to given budget	Always	131 (0.46)	168 (0.37)	299 (0.41)	0.056
	Usually	69 (0.24)	104 (0.23)	173 (0.23)	
	Sometimes	43 (0.15)	89 (0.20)	132 (0.18)	
	Never	43 (0.15)	89 (0.20)	132 (0.18)	
I spend whatever I have	Always	64 (0.22)	126 (0.28)	190 (0.26)	<0.0001
	Usually	53 (0.18)	100 (0.22)	153 (0.21)	
	Sometimes	55 (0.19)	118 (0.26)	173 (0.23)	
	Never	114 (0.40)	106 (0.24)	220 (0.30)	
I keep my savings in bank account	Always	46 (0.16)	95 (0.21)	141 (0.19)	<0.0001
	Usually	21 (0.07)	54 (0.12)	75 (0.10)	
	Sometimes	12 (0.04)	44 (0.10)	56 (0.08)	
	Never	207 (0.72)	257 (0.57)	464 (0.63)	

**Table-II:** Factor analysis of Financial Wellness

		**Financial Security**	**Care towards expenses**
Financial Wellness Items	Financial goals	0.795	
	Bank account	0.357	
	Restrict to budget		0.699
	Making budget		0.598
	Spend without thrift		-0.313
Institute Type	Public MC	2.50 ± 1.75	5.10 ± 2.13
	Private MC	2.94 ± 1.78	5.14 ± 2.07
	P Value	0.001	0.982

***Recommendations: ***All students need to be financially literate, focused in terms of planning of budget and restricting expenses within their means. The FW and FL knowledge borrowed from the business, finance and management world can be disseminated by research seminars and practice facilitated by budget charts and special software’s that may help to acquire the desired academic purpose at all health professional related academic institutes in our country. The final outcome of this research exercise will produce financial literate physicians, attain international standards and eventually build a progressive Pakistan, as a part of our desired MDGs.

## Authors’ Contribution:

***Dr. Rehana Rehman: ***Principal investigator: study design and overall supervision of the project.

***Dr. Shahjahan Katpar: ***Took part in acquisition of data, interpretation of data, drafting the article and revising it critically for important intellectual content.

***Dr. Rakhshaan Khan: ***Took part in compilation of data, drafting the article and revising it.

***Miss Mehwish Hussain: ***Statistician: Took part in data analysis, interpretation of data.
